# Supplementary material for: Hybrid Models and Biological Model Reduction with PyDSTool
Source: PLoS Comput Biol. 2012 Aug 9;8(8):e1002628. doi: 10.1371/journal.pcbi.1002628 (PMC3415397; doi:10.1371/journal.pcbi.1002628)
Supplement: Text S4 — Complete source code for the PyDSTool package (version 0.88.120504). Includes API documentation and help files linking to web pages. This file is identical to the current public release on Sourceforge.net. (ZIP) [file pcbi.1002628.s004.zip › PyDSTool/html/PyDSTool.MProject-module.html]

xml version="1.0" encoding="ascii"?


PyDSTool.MProject


| Home | Trees | Indices | Help | PyDSTool |
| --- | --- | --- | --- | --- |

Package PyDSTool ::
Module MProject

[hide private]

[frames] | no frames]

# Module MProject

source code

Modelling project and associated classes.

For aiding model estimation using (hybrid) dynamical systems.

Robert Clewley, September 2007.


|  |  |
| --- | --- |
| Classes | [hide private] |

ModelLibrary  
Store a set of related candidate model types, and within each,
represent various relevant "dimensions" along which the
model can be augmented structurally.

GenTransform  
Generator Transformer class.

ModelTransform  
Model Transformer class.

ModelManager  
Model management and repository class.

feature  
End users of concrete sub-classes provide (required) evaluate
method and (optional) prepare, finish methods.

feature\_leaf  
Abstract super-class for feature leaf nodes.

feature\_node  
Abstract super-class for feature regular nodes (supporting
sub-features).

ql\_feature\_leaf  
Qualitative feature (leaf node).

qt\_feature\_leaf  
Quantitative feature (leaf node).

ql\_feature\_node  
Qualitative feature (regular node).

qt\_feature\_node  
Quantitative feature (regular node).

condition  
Model context condition, made up of a boolean composition of wanted
and unwanted features.

context  
A collection of related model interfaces that apply to a model.

always\_feature  
Use this for a single vector field model that uses discrete event
mappings.

binary\_feature  
Use this as a binary switch feature, toggled by a given variable
name 'varname' that is supplied in the pars dict at initialization.

dsInterface  
Generic and abstract interface class for dynamical systems.

GeneratorInterface  
Wrapper for Generator (for non-hybrid models) that shares similar
API with ModelInterface for use in HybridModel objects.

ModelInterface  
Model constraints expressed as a uni-directional interface to another
formal system model:
- Made up of conditions imposed on the other system's test trajectory.

intModelInterface  
Interface providing internal evaluation criteria between models.

extModelInterface  
Interface from a trajectory of numerical data and test conditions
providing external evaluation criteria for a model.

MReg  
Registry class for Model descriptors and instances in Model
projects.


|  |  |
| --- | --- |
| Functions | [hide private] |

|  |  |
| --- | --- |
| extract\_from\_model(model) | source code |

|  |  |
| --- | --- |
| process\_raw\_residual(raw\_residual, weights) | source code |


|  |  |
| --- | --- |
| Variables | [hide private] |

\_classes = `['GenTransform', 'ModelTransform', 'ModelManager', ...`

\_functions = `['extract_from_model']`


|  |  |
| --- | --- |
| Variables Details | [hide private] |

### \_classes

Value:
:   ```
    ['GenTransform',
     'ModelTransform',
     'ModelManager',
     'feature',
     'ql_feature_node',
     'ql_feature_leaf',
     'qt_feature_node',
     'qt_feature_leaf',
    ...
    ```

  


| Home | Trees | Indices | Help | PyDSTool |
| --- | --- | --- | --- | --- |

|  |  |
| --- | --- |
| Generated by Epydoc 3.0.1 on Fri May 4 15:24:03 2012 | http://epydoc.sourceforge.net |
